# Differential Effects of Probiotic Strains on Chronic Stress-Exacerbated Colonic Motility in Rats: A Comparative Evaluation

**DOI:** 10.3390/metabo15100677

**Published:** 2025-10-19

**Authors:** Yun-Seong Lee, Soyu Lee, Sooah Kim

**Affiliations:** 1 Department of Microbiology and Immunology, School of Medicine, Wonkwang University, Iksan 54538, Republic of Korea; iyoonseong@daum.net; 2 Nain Healthcare Co., Ltd., Iksan 54613, Republic of Korea; 3 Department of Conservative Dentistry, School of Dentistry, Jeonbuk National University, Jeonju 54896, Republic of Korea; 4 Department of Environment Science & Biotechnology, Jeonju University, Jeonju 55069, Republic of Korea

**Keywords:** probiotics, strain specificity, irritable bowel syndrome, water avoidance stress, gut–brain axis

## Abstract

Background/Objectives: Psychological stress is a main factor in the pathophysiology of irritable bowel syndrome (IBS) and contributes to changes in gastrointestinal motility and inflammatory responses. We investigated the effects of three probiotic strains, *Lactobacillus brevis* N1, *L. brevis* N2, and *Bacillus amyloliquefaciens* S1, isolated from Korean fermented foods, on stress-induced colonic hypermotility and inflammatory signaling in a rat model. Methods: Thirty female Wistar rats were divided into five groups: Control (sham), Stress (water avoidance stress, WAS), Treatment A (WAS + *L. brevis* N1), Treatment B (WAS + *L. brevis* N2), and Treatment C (WAS + *B. amyloliquefaciens* S1) (*n* = 6 per group). Rats were exposed to WAS for 1 h daily for nine consecutive days. Furthermore, before stress exposure, probiotics were administered by oral gavage. The fecal pellet output (FPO), body weight, and food intake were recorded daily. Colon tissues were harvested for protein extraction, and inflammatory signaling was evaluated by Western blotting for NF-κB and IκBα, with β-actin as loading control. Immunoreactive bands were visualized by enhanced chemiluminescence (ECL) and quantified using ImageJ software version 1.54k. Results: The WAS group showed significantly higher FPO than the sham group (*p* < 0.01). FPO was significantly decreased in rats treated with *L. brevis* N2 compared to that in the WAS-only group (*p* < 0.05). Additionally, immunohistochemical analysis revealed that NF-κB expression was suppressed in all the probiotic groups. Conclusions: Therefore, probiotics are suggested to have elevated anti-inflammatory effects through the downregulation of the NF-κB signaling pathway by restoring IκBα expression and can be utilized as potential therapeutics for stress-induced gastrointestinal dysfunction.

## 1. Introduction

Irritable bowel syndrome (IBS) is a common functional gastrointestinal disorder characterized by recurrent abdominal pain and altered bowel features without structural abnormalities [1]. Psychological stress has consistently been implicated as a main aggravating factor in IBS pathophysiology, despite its complex and multifactorial etiology [2]. Preclinical and clinical studies have revealed that chronic psychological stress can disrupt gastrointestinal motility and sensation by activating the brain–gut axis (BGA), a bidirectional communication network linking the central and enteric nervous systems via neural, hormonal, and microbial signaling [3,4]. The water avoidance stress (WAS) model has been widely used as an experimental model to induce colonic dysmotility in rodents and mimic characteristics similar to stress-sensitive IBS in humans [5]. WAS considerably increases fecal pellet output (FPO) and colonic transit through the activation of the corticotropin-releasing factor (CRF) signaling pathway in the central nervous system [6,7]. Probiotics have gained traction as a potential therapy for IBS since they improve gut motility, enhance epithelial barrier integrity, and regulate visceral sensitivity [8,9,10]. However, some studies have reported that probiotics’ beneficial effects are strain dependent and not all strains exhibit equal efficacy against stress-induced gastrointestinal dysfunction [11,12]. Furthermore, certain strains of *Lactobacillus*, *Bifidobacterium*, and *Bacillus* species can alleviate IBS symptoms [13,14,15]. For example, *L. plantarum* and *B. coagulans* decrease the colonic transit time and normalize motility patterns in models of functional bowel disorders [16,17]. However, the basic mechanisms responsible for the strain-specific effects under stress-induced hypermotility conditions remain elusive.

The nuclear factor kappa-light-chain-enhancer of activated B cells (NF-κB) signaling pathway is dysregulated in different diseases such as inflammatory bowel disease, cancer, and rheumatoid arthritis [18]. The gut microbiota consists of a complex microorganism community and plays a crucial role in regulating immune and inflammation function by modulating the NF-κB signaling pathway [19]. Under stress, microbiota dysbiosis is induced, resulting in increased intestinal permeability, which allows microbial products, such as lipopolysaccharide, to cross the barrier. These products activate NF-κB via pattern recognition receptors, such as toll-like receptors (TLRs), triggering inflammatory cascades [20,21]. Additionally, psychological stress disrupts the gut microbiota and promotes NF-κB-mediated inflammation [22]. Probiotics are live microorganisms that confer health benefits and have been used to regulate the gut microbiota and reduce inflammation [23]. Probiotic-specific strains such as *L. reuteri* can inhibit NF-κB signaling pathway activation by preventing the degradation of its inhibitors, resulting in the promotion of anti-inflammatory function [24] and restoration of microbiota composition for increasing beneficial genera, including Bacteroides and Parabacteroides [25].

**Figure 1:**
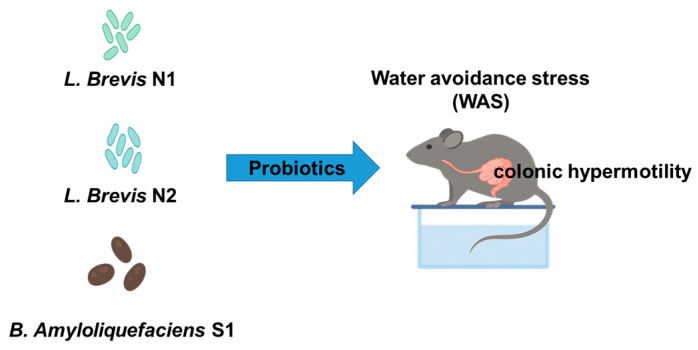
Strain-specific modulation of stress-induced colonic hypermotility by probiotics in a rat model.

Herein, we investigated the modulatory effects of three distinct probiotic strains, *L. brevis* N1, *L. brevis* N2, and *B amyloliquefaciens* S1, isolated from traditional Korean fermented foods, on chronic psychological stress-induced colonic hypermotility in Wistar rats using the WAS model (Figure 1).

## 2. Materials and Methods

### 2.1. Animal Experiments

Thirty female Wistar rats (6 weeks old, 180–220 g) were purchased from Orient Bio, Inc. (Seoul, Republic of Korea). Each rat was individually housed in a standard cage. The environmental conditions of the cages were kept at 23 ± 2 °C, at 50 ± 10% humidity, and a 12-h light/dark cycle. The Wonkwang University Animal Experimental Ethics Committee approved the animal experiments (approval no. WKU22-130).

### 2.2. Experiment Design

After acclimation for one week, rats were randomly assigned to five groups using a computer-generated random sequence to ensure unbiased group allocation: Control (No-stress), Stress (WAS only), Treatment A (WAS with *L. brevis* N1), Treatment B (WAS with *L. brevis* N2), and Treatment C (WAS with *B. amyloliquefaciens* S1), which were isolated from Korean fermented food (Table 1).

**Figure 2:**
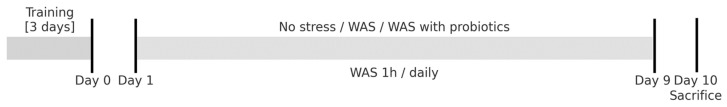
Experimental design.

Rats in the stress group were exposed to WAS daily for 1 h for 9 days, whereas rats in the non-stress group were exposed to sham WAS (Figure 2). Vehicles or probiotics were administered by oral gavage before WAS. The probiotics were suspended in 10% (*w*/*v*) skim milk at a final concentration of 1 × 10^9^ CFU (Colony-Forming Unit).

### 2.3. Physiological Measurements

Body weight and food intake were recorded daily throughout the 9-day experimental period. Food intake was calculated as the difference between the pre-weighed chow amount and the amount remaining after 24 h. FPO, an index of colonic motility, was measured by collecting and counting fecal pellets during each 1-h WAS session. The total number of pellets expelled was recorded for each animal, and the cumulative FPO over 9 days was calculated.

### 2.4. Microbiome Analysis

Fecal samples were collected from each animal before and after stress application. Fecal samples were collected from each animal and immediately stored at −80 °C until DNA extraction. Microbial community profiling was performed by 16S rRNA gene sequencing. DNA was extracted from fecal samples using a QIAamp DNA Stool Mini Kit (Qiagen, Hilden, Germany). The yield and purity of the DNA were assessed using a NanoDrop spectrophotometer (Thermo Fisher Scientific, Waltham, MA, USA), confirming adequate concentration and A_260/280 ratios of ~1.8–2.0. The V3–V4 hypervariable region of the 16S rRNA gene was amplified using universal primers and sequenced on an Illumina MiSeq platform (Illumina, San Diego, CA, USA). Sequence reads were processed using QIIME2 (2023.2), with operational taxonomic units (OTUs) clustered at 97% similarity and taxonomy assigned using the SILVA 16S rRNA database. Beta diversity patterns were visualized using non-metric multidimensional scaling (NMDS) plots based on the Bray–Curtis dissimilarity. In the plots, the arrows indicate the directionality of the taxonomic shifts, and ellipses represent 95% confidence intervals for the distribution of each group.

### 2.5. Tissue Collection and Protein Extraction

Colon tissues were harvested from rats in each experimental group for the assessment of inflammatory signaling protein expression. Tissues were homogenized in radioimmunoprecipitation assay (RIPA) buffer (Thermo Fisher Scientific, Waltham, MA, USA) containing protease and phosphatase inhibitors. Homogenates were incubated on ice for 60 min and centrifuged at 12,000 rpm for 30 min at 4 °C. The supernatants were collected, and protein concentrations were determined using a bicinchoninic acid (BCA) protein assay kit (Thermo Fisher Scientific, Waltham, MA, USA).

### 2.6. Western Blot Analysis

Equal amounts of protein (30 µg) were mixed with Laemmli sample buffer, denatured and separated on 10% SDS–polyacrylamide gels. Proteins were transferred to polyvinylidene fluoride (PVDF) membranes (Millipore, Burlington, MA, USA) using a semi-dry transfer system for 60 min. Membranes were blocked with 5% skim milk in TBS-T (20 mM Tris-HCl, 150 mM NaCl, 0.05% Tween-20, pH 7.5) for 1 h at room temperature. Membranes were incubated overnight at 4 °C with the following primary antibodies for Western blotting were used as follows: anti-NF-κB p65 (rabbit mAb, Cell Signaling Technology, Danvers, MA, USA, #8242) at 1:1000 dilution; anti-IκBα (rabbit polyclonal, Cell Signaling Technology, Danvers, MA, USA, #9242) at 1:1000; and anti-β-actin (mouse mAb, Cell Signaling Technology, Danvers, MA, USA, #3700) at 1:2000. After washing with TBS-T, membranes were incubated with horseradish peroxidase (HRP)-conjugated secondary antibody (goat anti-rabbit IgG, 1:3000; Calbiochem, Darmstadt, Germany) for 1 h at room temperature. Immunoreactive bands were visualized using an enhanced chemiluminescence (ECL) detection kit (EZ-Western Lumi Femto, DoGen, Seoul, Republic of Korea) and quantified using ImageJ software version 1.54k (National Institutes of Health, Bethesda, MD, USA).

### 2.7. Tissue Collection and Fixation

Colonic segments were harvested from rats in each experimental group: Control, Stress (WAS only), Treatment A, B, and C immediately following euthanasia. Samples were fixed in 10% neutral-buffered formalin for 24 h, dehydrated using graded alcohols, cleared in xylene, and embedded in paraffin. Serial 4–5 μm sections were cut and mounted on poly-L-lysine-coated slides.

### 2.8. Immunohistochemistry (IHC) Analysis

The slides were deparaffinized, rehydrated, and subjected to antigen retrieval using 10 mM citrate buffer (pH 6.0). Endogenous peroxidase activity was blocked using 3% hydrogen peroxide. Furthermore, the sections were incubated overnight at 4 °C with anti-NF-κB p65 antibody (1:100 dilution; Cell Signaling Technology, Danvers, MA, USA, #8242), followed by a biotinylated secondary antibody and streptavidin–HRP. Color development was achieved using the DAB substrate, and the slides were counterstained with hematoxylin [26]. Tissue sections were stained and observed under a bright-field microscope (Olympus BX53, Tokyo, Japan).

### 2.9. Physiological Measurements

Statistical analyses were performed using GraphPad Prism 9.0 (GraphPad Software, San Diego, CA, USA). All data were expressed as the mean ± standard deviation. Intergroup comparisons were performed using one-way analysis of variance, followed by Tukey’s post hoc test for multiple comparisons. Statistical significance was set at *p* < 0.05.

## 3. Results

### 3.1. Body Weight and Food Intake

Daily monitoring of body weight and food intake showed no statistically significant differences among the experimental groups over the course of the 9-day experiment. All rats revealed a progressive elevation in body weight, indicating a healthy physiological status and the absence of adverse effects from either the WAS or probiotic supplementation.

**Figure 3:**
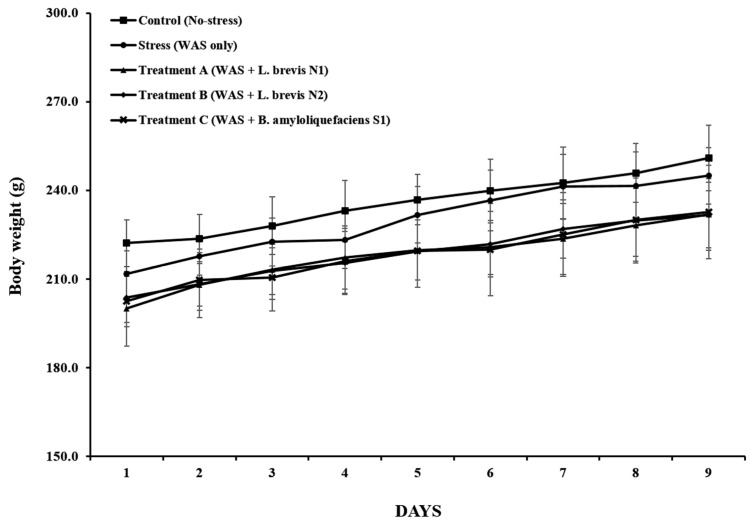
Alterations in body weight over the 9-day experimental period in rats exposed to water avoidance stress (WAS) and/or probiotic treatment. The data are expressed as the mean ± standard deviation (*n* = 6 per group).

Body weight steadily increased in all groups exhibited weight gain (Figure 3). The final body weight was 212.3 ± 5.4 g in the control group and 207.8 ± 4.9 g in the stress group, with this difference not being statistically significant (*p* = 0.31). Similarly, final body weights in Treatment A, B, and C were 209.5 ± 5.1 g, 211.0 ± 4.8 g, and 208.6 ± 5.3 g, respectively (all *p* > 0.05 vs. Stress).

**Figure 4:**
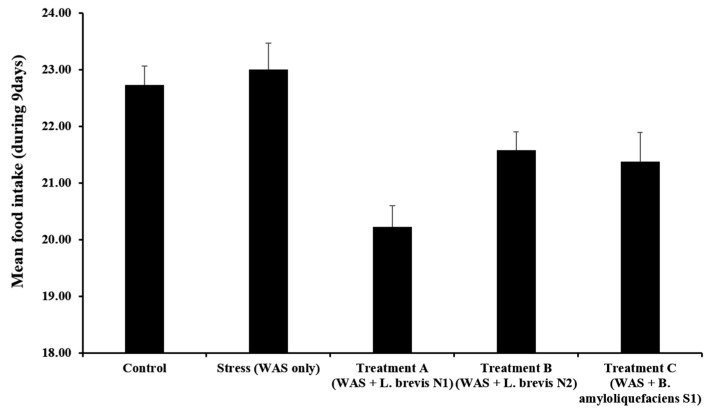
Mean of the food intake during the 9-day experimental period in rats subjected to WAS and probiotic intervention. The data represent the mean ± standard deviation (*n* = 6 per group). Control (no stress, vehicle only); Stress (WAS only); Treatment A (WAS + *Lactobacillus brevis* N1); Treatment B (WAS + *Lactobacillus brevis* N2); Treatment C (WAS + *Bacillus amyloliquefaciens* S1).

All groups revealed relatively consistent food intake levels, with no significant differences (Figure 4). The stress group consumed 21.3 ± 1.2 g/day of chow on average, compared to 20.5 ± 1.0 g/day in the control group (*p* = 0.45). The probiotic-treated groups showed mean food intakes of 19.4 ± 1.3 g/day (Treatment A), 20.8 ± 1.1 g/day (Treatment B), and 19.9 ± 1.2 g/day (Treatment C), with no significant differences versus the stress group (*p* > 0.05 for all). Notably, Treatment B group exhibited slightly lower food intake than the WAS-only group; however, this was higher than that in the other probiotic groups, suggesting that this strain could help stabilize feeding behavior under stress conditions.

Taken together, these findings indicate that the WAS model does not significantly affect general metabolism or feeding behavior. Additionally, the probiotic treatment did not adversely affect body weight or food intake. Thus, the differences in FPO between the groups were mainly attributable to the direct regulation of gut motility rather than the alterations in nutritional status or energy balance.

### 3.2. Daily FPO

FPO was calculated daily during each 1-h WAS to assess the effects of stress and probiotic administration on colonic motility. Rats in the stress group showed a substantial increase in colonic motility compared with those in the control group. The mean FPO of the stress group was significantly increased (6.56 ± 1.42 pellets) compared to the control group (0.83 ± 1.01 pellets) (*p* < 0.01).

These results demonstrate the WAS model’s effectiveness in inducing stress-associated hypermotility. Among the probiotics groups, the group treated with Treatment B revealed a significantly reduced number of FPO compared to the stress group (3.89 ± 0.95 vs. 6.56 ± 1.42, *p* < 0.05). This suggests a strain-specific protective effect of the Treatment B group on colonic activity under stressful conditions. Contrastingly, the groups treated with Treatment A and Treatment C showed the mean FPO values of 4.79 ± 1.11 and 5.11 ± 1.23 pellets, respectively, which were lower than that in the stress group with no significant difference (*p* > 0.05).

**Figure 5:**
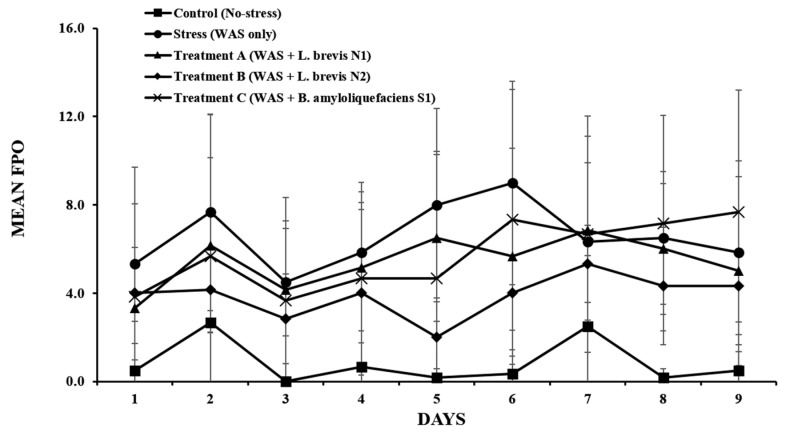
Daily fecal pellet output (FPO) over 9 days in rats subjected to water avoidance stress (WAS) and/or probiotic treatment. The data represent the mean ± standard deviation (*n* = 6 per group).

The FPO patterns under stress and probiotic treatments were clearly different. The FPO levels remained low in the control group, whereas the stress group revealed increased colonic motility. The Treatment B group showed a reduction in FPO starting from day 4 compared to the stress group, suggesting that repeated administration of *L. brevis* N2 could have a beneficial effect on gut motility over time (Figure 5). 

Thus, *L. brevis* N2 has a beneficial effect on the stress-induced increase in colonic motility in rats. Contrastingly, other probiotic strains such as *L. brevis* N1 and *B. amyloliquefaciens* S1 revealed no noticeable effects under the same experimental conditions.

### 3.3. Accumulated FPO

**Figure 6:**
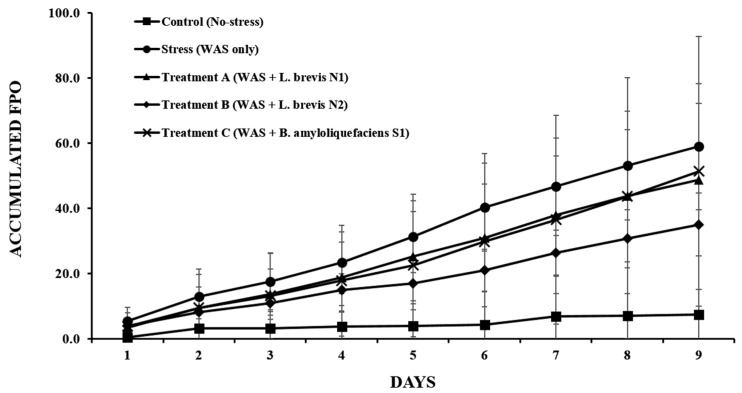
Cumulative fecal pellet output (FPO) over the 9-day water avoidance stress (WAS) exposure period. The data are expressed as the mean ± standard deviation (*n* = 6 per group).

The accumulated FPO, as illustrated in Figure 6, differed between the groups, indicating a difference in colonic motility. This value in the stress group rapidly increased compared to that in the control group. Furthermore, the accumulated FPO of the stress group was higher than that of the other groups over 9 days, indicating a strong hypermotility response caused by chronic psychological stress.

The group treated with Treatment B revealed a reduction in accumulated FPO compared to the stress group. After four days, the separation of these groups, such as Treatment B and stress groups, was initiated and continued during the experimental period. Although the difference was not significant every day, the higher *L. brevis* pattern suggested that repeated administration in the Treatment B group could result in beneficial effects in stabilizing colonic motility under stress conditions. Other probiotics, such as Treatments A and C groups in the accumulated FPO, were reduced compared with the stress group. However, this effect was less pronounced than that observed in the Treatment B group. Consistent with the daily FPO findings, these results showed that Treatment B could alleviate stress-induced colonic hypermotility, and repeated administration of probiotics, such as in the Treatment B group, exerted adaptive effects on the gut motor function.

**Figure 7:**
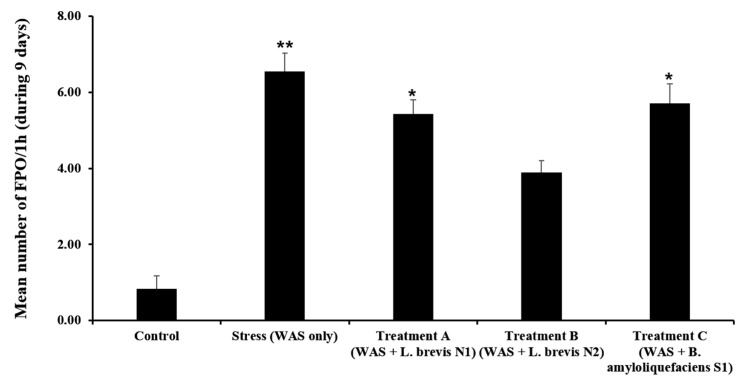
Mean number of fecal pellet output (FPO) per hour during the 9 days. The data are presented as the mean ± standard deviation (*n* = 6 per group). * *p* < 0.05, ** *p* < 0.01 vs. Control group.

Finally, we calculated the mean FPO number over nine days to assess the overall effects of probiotics on colonic motility (Figure 7). The FPO number in the stress group expelled a cumulative total of 58.3 ± 5.2 fecal pellets per mouse, which was significantly higher than the 7.5 ± 2.1 pellets in the control group (*p* < 0.001). Probiotic treatments attenuated this stress-induced increase in cumulative FPO to varying degrees. The Treatment A group had a 9-day total of forty-three (43.0 ± 4.5) pellets, and the Treatment C group had 47.8 ± 5.0 pellets; both values were lower than that of the stress group but did not reach statistical significance (*p* > 0.05 vs. Stress). Notably, the Treatment B group showed a cumulative FPO of 34.6 ± 6.1 pellets, which was significantly lower than the stress group (*p* = 0.04). These results indicate that chronic stress markedly elevates cumulative fecal output, and among the probiotic interventions, *L. brevis* N2 (Treatment B) was the most effective in reducing this elevated colonic motility.

### 3.4. Gut Microbiota Shifts

**Figure 8:**
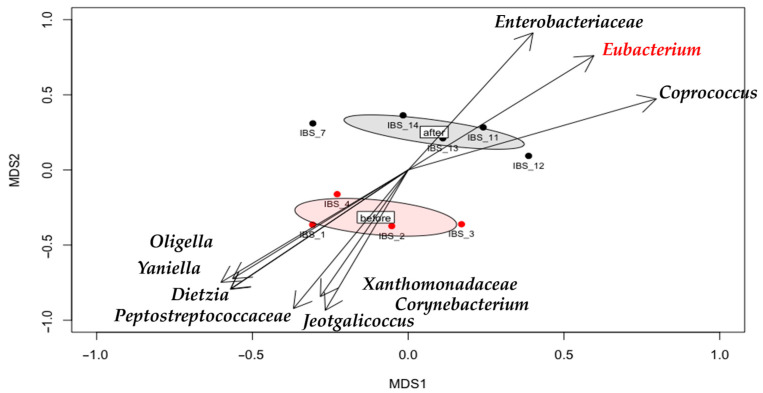
NMDS plot illustrating the compositional changes in the gut microbiota in IBS model rats before (red) and after (black) probiotic treatment. Each point represents an individual rat. Arrows indicate the directional shift in the microbial community composition after intervention. Ellipses represent 95% confidence intervals for each group. The “before” state was enriched in the opportunistic bacteria such as *Oligella*, *Corynebacterium*, and *Peptostreptococcaceae*, whereas the “after” state revealed a restoration toward beneficial taxa, including *Bacteroides*, *Parabacteroides*, *Roseburia*, and *Mucispirillum*.

In this analysis, “before” refers to the baseline microbiota composition assessed prior to WAS exposure and prior to probiotic treatment whereas “after” refers to the microbiota composition at the end of the 9-day WAS exposure after probiotic treatment in the respective groups. The NMDS plots (Figure 8) show distinct clustering of the microbial communities at these two time points. In the “before” state, the gut microbiota was dominated by potentially pathogenic or stress-related taxa (e.g., *Oligella*, Corynebacterium, *Peptostreptococcaceae*), whereas in the “after” state following stress and probiotic intervention there was a notable increase in beneficial taxa such as *Bacteroides*, *Parabacteroides*, *Roseburia*, and *Mucispirillum* especially in probiotic-treated groups. The directional vectors on the NMDS plot illustrate the trajectory of microbial community shift from the dysbiotic “before” state to a more eubiotic “after” state in the presence of probiotic treatment. Alpha diversity was calculated and showed no significant differences between groups or between the before and after samples (*p* > 0.05), indicating that within-sample diversity remained relatively unchanged despite the compositional shifts.

### 3.5. NF-κB and IκBα Protein Expression

**Figure 9:**
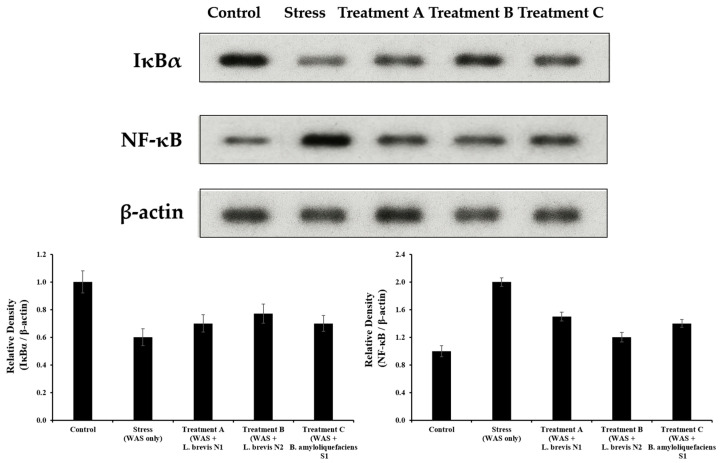
Protective effects of probiotic treatments on stress-induced NF-κB signaling pathway in rat. Western blot analysis of IκBα and NF-κB protein expression levels in the colon tissues (upper panels). Densitometric quantification of IκBα/β-actin (left) and NF-κB/β-actin (right) ratios. Stress (WAS only) markedly decreased IκBα and increased NF-κB expression compared with the control group. Probiotic treatments (*Lactobacillus brevis* N1, *Lactobacillus brevis* N2, and *Bacillus amyloliquefaciens* S1) restored IκBα expression and attenuated NF-κB activation. Data are expressed as the mean ± SD.

Western blot analysis revealed that WAS (stress) induction significantly upregulated NF-κB expression while downregulating its inhibitory protein, IκBα, compared with the control group (*p* < 0.01). This molecular signature indicates NF-κB pathway activation through IκBα degradation and nuclear translocation of NF-κB. Probiotic administration (*L. brevis* N1, *L brevis* N2, *B. amyloliquefaciens* S1). significantly reversed these alterations. NF-κB protein levels were reduced, while IκBα levels were restored compared with the WAS group (*p* < 0.05). Among the treatments, the Treatment B group exhibited the most pronounced effect, with IκBα expression approaching near-control levels (Figure 9). These findings are in strong agreement with the study by Khodabakhsh et al. who reported that Montelukast treatment suppressed NF-κB expression and reduced pro-inflammatory cytokines in IBS rats, highlighting the canonical role of NF-κB signaling in stress-induced gut inflammation [27].

### 3.6. IHC Analysis

**Figure 10:**
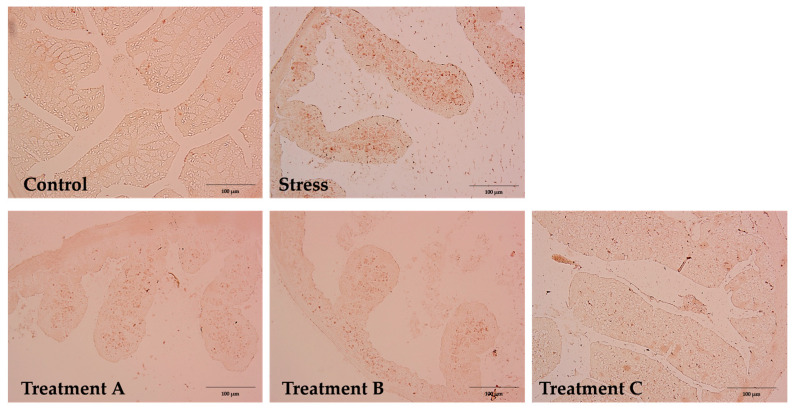
Representative IHC staining for NF-κB p65 in colonic mucosa from a rat. NF-κB expression is minimal, and the tissue architecture is intact. Brown nuclei indicate NF-κB localization. Sections were counterstained with hematoxylin. Scale bar = 100 μm. Magnification: 100×. Control (no -stress, vehicle only); Stress (WAS only); Treatment A (WAS + *Lactobacillus brevis* N1); Treatment B (WAS + *Lactobacillus brevis* N2); Treatment C (WAS + *Bacillus amyloliquefaciens* S1).

NF-κB expression was minimal in the control group, with only weak and localized nuclear staining (Figure 10). The tissue structure remained intact with no signs of inflammation. Contrastingly, the stress group showed a significant increase in NF-κB-positive nuclei, especially in the epithelial layer and lamina propria, indicating stress-induced inflammatory activation (*p* < 0.05) [28].

Probiotic-treated groups showed a dose-dependent reduction in NF-κB expression. Treatment A revealed the most prominent effect, restoring the staining pattern to near-normal levels and significantly decreasing the number of positive nuclei compared with the WAS group (v). Although Treatments B and C also showed a reduction in the NF-κB staining, their effects were relatively less pronounced than those of Treatment A.

## 4. Discussion

This study confirmed that chronic psychological stress induced by WAS increased colonic motility. Stress-induced responses were regulated in a specific strain by probiotic treatment. We compared three strains, *L. brevis* N1, *L. brevis* N2, and *B. amyloliquefaciens* S1, and found that *L. brevis* N2 demonstrated the greatest effect on stress-induced colonic hypermotility. These findings are consistent with those of a previous study revealing that psychological stress is a strong contributor to gastrointestinal dysfunction, especially associated with the motility alterations observed in IBS [29]. Under stress, the CRF system is activated and the hypothalamic–pituitary–adrenal axis is triggered, ultimately resulting in enhanced colonic transit via parasympathetic activation [30]. The WAS model used in this study has been widely validated as a reliable experimental method for reproducing chronic stress-induced gastrointestinal symptoms in rodents, such as hyperdefecation and visceral hypersensitivity [31]. Differences in probiotic efficacy have been recognized to be highly strain-specific. Although different *Lactobacillus* species share similar genomic traits, the effects of each strain may differ on host physiology [32]. In particular, FPO was consistently reduced in rats treated with *L. brevis* N2, revealing that repeated probiotic administration may be necessary for colonization in the gut and interaction with the neuronal system [33]. The BGA is a two-way networking system consisting of neural, endocrine, and immune signaling pathways regulated by the enteric nervous system, vagus nerve, and metabolites produced by the gut microbiota. Therefore, it functions as a key pathway through which probiotics exert their effects [34]. Probiotics can regulate vagal afferent signaling, produce neuroactive compounds such as serotonin and γ-aminobutyric acid, and influence enteric neurotransmission via modifications in the epithelial barrier integrity or mucosal function [35,36]. In the present study, *B. amyloliquefaciens* S1, known for its anti-inflammatory properties, did not substantially influence colonic motility under stress conditions. While this strain can reveal immunomodulatory effects under other conditions, its lack of effect on motility in this model suggests that it may not sufficiently interact with the enteric motor system or the neuroendocrine axis activated by stress [37]. Certain probiotic strains have been shown to normalize stress by inducing increased CRF expression, suppressing vagal-sympathetic imbalance, and modulating downstream targets, such as serotonin transporters and mucosal nerve endings [38,39].

Consistent with previous studies, the findings of the present study indicated that psychological stress, such as WAS, activates the NF-κB signaling pathway in the colon and contributes to an increase in inflammation, which is a pathological feature of IBS [40]. NF-κB is a key transcriptional regulator of pro-inflammatory cytokines such as TNF-α, IL-6, and IL-1β [41]. Stress-induced IBS is characterized by NF-κB p65 overexpression and IκBα degradation, which drive the transcription of inflammatory cytokines such as TNF-α and IL-1β [42,43]. NF-κB expression in the colonic mucosa has been shown to increase in animal models of IBS and humans [44]. In this study, a slight discrepancy was observed between molecular and histological assessments. *L. brevis* N2 had the most pronounced effect in reducing NF-κB activation according to Western blot restoring IκBα and lowering NF-κB levels, whereas *L. brevis* N1 showed the strongest suppression of NF-κB positive cells in the IHC analysis. This may be due to differences in detection methods Western blot measures total protein levels in tissue homogenates, while IHC highlights cell-specific nuclear localization of NF-κB suggesting that *L. brevis* N1 might more effectively prevent NF-κB nuclear translocation in certain cell types even though *L. brevis* N2 provides a greater overall reduction in NF-κB pathway activity. In the present study, the suppression of the NF-κB expression in the probiotic groups suggested that probiotic treatment can increase anti-inflammatory effects through NF-κB activity inhibition. A similar effect was observed in a study, where natural compounds such as baicalein alleviated visceral hypersensitivity in rats by downregulating NF-κB activation [45]. Additionally, another study reported that the traditional herbal formula sinisan inhibits colonic TLR4/MyD88/NF-κB signaling in IBS models [46]. Furthermore, the strain-specific efficacy observed in the WAS+N1 group indicates that probiotics may differentially modulate upstream regulators of NF-κB, akin to drug-mediated blockade of inflammatory pathways. Collectively, these results establish a coherent model in which probiotics alleviate IBS symptoms by preventing IκBα degradation, thereby inhibiting NF-κB p65 activation and suppressing downstream inflammatory cascades [47]. Our IHC staining results support NF-κB’s role as a core inflammatory mediator in IBS and highlight the therapeutic potential of targeting NF-κB via probiotic interventions. This study provides a broader understanding of how stress-induced inflammation can be modulated at the molecular and microbial levels by integrating histological analysis with microbiota profiling. Nevertheless, we acknowledge several limitations of this study. The use of the WAS rat model, while well-established for simulating IBS-like stress responses, may not encompass the full complexity of human IBS. The experiment was of relatively short 9 days of stress, whereas IBS in humans is a chronic condition, so longer-term studies would be informative. We also focused on colonic motility and NF-κB signaling, and did not directly measure other potentially relevant factors. In particular, upstream TLR signaling and cytokine profiling should be included to provide a deeper mechanism into the regulation of NF-κB signaling pathway. Future studies should investigate these probiotic strains in other IBS models or clinical trials to determine if the strain-specific benefits translate to humans. In addition, we have to elucidate the mechanisms behind their anti-inflammatory and motility-regulating effects such as by examining cytokine profiles, stress hormone levels, or GBA signaling in probiotic-treated subjects. Moreover, the integrated analysis of microbiome metabolites using multi-omics approaches should be conducted to provide functional relationships between gut microbial changes and metabolic pathways.

## 5. Conclusions

In this study, we demonstrated that chronic psychological stress induced by WAS significantly increased colonic motility and activated inflammatory signaling in a rat model of IBS. Among the three probiotic strains tested including *L. brevis* N1, *L. brevis* N2, and *B. amyloliquefaciens* S1, *L. brevis* N2 showed the highest suppressive effect on stress-induced colonic hypermotility. This strain restored IκBα expression and suppressed NF-κB activity, supporting its anti-inflammatory potential. These findings suggested that the specific probiotics can be used as the therapeutic potential in stress-induced gastrointestinal dysfunctions such as IBS.

## Figures and Tables

**Table 1:** Experimental design of the animal study for 9 days to assess the avoidance stress effects of probiotics.

Group	Material	Dose	*n*
Control (No-stress)	negative control (10% skim milk)	-	6
Stress (WAS only)	negative control (10% skim milk)	-	6
Treatment A	WAS + *Lactobacillus brevis* N1	1 × 10^9^ CFU	6
Treatment B	WAS + *Lactobacillus brevis* N2	1 × 10^9^ CFU	6
Treatment C	WAS + *Bacillus amyloliquefaciens* S1	1 × 10^9^ CFU	6

## Data Availability

Dataset available on request from the authors.
